# Clinical Efficacy of Topical Ascorbic Acid 2-Glucoside in Enhancing Outcomes of Laser Gingival Depigmentation: A Randomized Controlled Trial

**DOI:** 10.7759/cureus.107127

**Published:** 2026-04-15

**Authors:** Tejasri Gudur, Sreenivas Nagarakanti, Sravya Sri Prudhvi, Pavithra Damai, Supriya Rupavath, Sreekar Lebaka

**Affiliations:** 1 Department of Periodontology, Narayana Dental College & Hospital, Nellore, IND

**Keywords:** ascorbic acid therapy, depigmentation, gingival hyperpigmentation, laser treatment, vitamin c

## Abstract

Background

Melanin accumulation in the oral epithelium causes gingival hyperpigmentation, which is a common aesthetic problem. Precision, low invasiveness, and favorable recovery make diode laser depigmentation (LD) the ideal method. Yet, recurrence of pigmentation is still a concern. The antioxidant and melanin inhibitor ascorbic acid 2-glucoside (AS-G) may improve results and postpone repigmentation. The effectiveness of topical AS-G following laser gingival depigmentation (LD + AS-G) was assessed in this study.

Materials and methods

A randomized controlled trial was conducted on 20 participants with physiologic gingival hyperpigmentation. Gingival depigmentation was performed in both LD + AS-G (test group) and LD (control group). Evaluations of clinical parameters, such as Dummett Oral Pigmentation Index (DOPI), Plaque Index (PI), and bleeding on probing, were performed at baseline, followed by assessments at one, three, six, and nine months. The Visual Analogue Scale (VAS) (pain and discomfort) was recorded on the seventh day, and VAS (satisfaction) was measured at six and nine months.

Results

To summarize, there was a significant reduction in PI within both groups over time, changes were observed in the modified Sulcus Bleeding Index (mSBI), intergroup differences were noted in DOPI scores at the nine-month follow-up, and differences were found in patient satisfaction scores at six and nine months. Intragroup analysis showed a statistically significant reduction in PI in both test and control groups at three, six, and nine months (p < 0.05). No significant changes were observed in the mSBI values in the test group, whereas the control group demonstrated a significant reduction only at nine months (p < 0.05). Intergroup comparison revealed no statistically significant differences in PI and mSBI scores at most follow-up intervals; however, a statistically significant difference in PI was observed at the nine-month follow-up (p = 0.022). DOPI scores demonstrated a statistically significant difference between the groups at nine months (p = 0.004), indicating higher recurrence of pigmentation in the control group. Patient satisfaction scores were comparable at six months but were significantly higher in the test group at nine months (p < 0.001).

Conclusions

Within the limitations of this randomized controlled trial, topical application of AS-G demonstrated beneficial effects in improving clinical outcomes following laser gingival depigmentation. The adjunctive use of this antioxidant appeared to enhance gingival healing and may contribute to improved patient-reported outcomes. These findings suggest that topical AS-G may serve as a useful adjunct in depigmentation procedures. Nevertheless, further studies with larger sample sizes and longer follow-up periods are recommended to validate these findings and establish long-term clinical benefits.

## Introduction

The study of aesthetics focuses on appreciating beauty. Therefore, the goal of aesthetic dentistry is to balance form and function while considering the individual preferences of each patient. The appearance of the teeth and gingiva plays a major role in creating an attractive smile. Tooth form, position, and shade, as well as gingival color and contour, all affect smile harmony [1]. Melanocyte activity, vascularization, and epithelial thickness and keratinization all affect gingival color [2]. Melanocyte activation causes melanin pigmentation, which can be influenced by genetic, racial, hormonal, systemic, or environmental factors, including smoking. This can lead to either pathological or physiological hyperpigmentation [3]. Therefore, a thorough medical evaluation is crucial [4].

Many patients, particularly those with gummy smiles, find physiological pigmentation unsightly, even if it is not pathogenic [5]. Among the various techniques for depigmentation, lasers are preferred for shorter treatment time, hemostasis, less invasiveness, and improved patient comfort. Regardless of the procedure and treatment mechanism used, repigmentation can occur. Ascorbic acid, or vitamin C, was discovered in 1912 and is essential for tissue repair and collagen formation [6]. It has been used as a depigmenting agent and inhibits tyrosinase, thereby lowering melanin synthesis. Despite the paucity of clinical studies, research in dentistry and dermatology has shown that it can delay repigmentation and reduce gingival pigmentation. Oral application has been enhanced by stabilized derivatives such as ascorbic acid 2-glucoside (AS-G) [7]. Hence, the current study aimed to evaluate the efficacy of topical AS-G on depigmented gingiva using a diode laser in delaying gingival repigmentation over a nine-month follow-up period.

## Materials and methods

Study design

This randomized controlled trial was conducted at the Department of Periodontology, Narayana Dental College & Hospital, Nellore, India, to evaluate the recurrence of physiological melanin pigmentation following gingival depigmentation using laser depigmentation (LD) alone and laser combined with topical AS-G (LD + AS-G). The study was carried out between February 2025 and November 2025. Ethical approval was obtained from the Institutional Ethics Committee (IEC/NDCH/2023/MAY/P-13), and the trial was registered with the Clinical Trials Registry - India (CTRI no.: CTRI/2025/01/079675). The study adhered to the ethical principles of the World Medical Association Declaration of Helsinki (1975, revised 2013) and was designed and reported in accordance with the CONsolidated Standards Of Reporting Trials (CONSORT) guidelines (2025). Written informed consent was obtained from all participants prior to enrollment.

Participants

Participants were recruited from patients attending the Department of Periodontology. Individuals were included if they were systemically healthy, aged 18 years or older, presented with physiologic gingival melanin pigmentation, and expressed aesthetic concern regarding gingival discoloration. Participants were excluded if they had pathologic pigmentation, periodontal disease or gingivitis, tobacco use, prior gingival depigmentation procedures, allergy to vitamin C, or any condition that could interfere with informed consent or compliance.

Sample size calculation

Sample size was determined using the formula for comparison of independent groups:

$n = \frac{2\left(Z_{\alpha/2} + Z_{\beta}\right)^2}{d^2},$

where Zα/2 represents the standard normal value for the significance level (1.96 for α = 0.05), Zβ represents the standard normal value corresponding to a statistical power of 80% (0.84), and d represents the effect size. Based on calculations performed using G*Power with an effect size of 1.25, a significance level of 0.05, and a statistical power of 80%, a minimum sample size of 18 participants (nine per group) was required. To account for an anticipated attrition rate of approximately 10%, the final sample size was increased to 20 participants, with 10 participants allocated to each group.

Randomization and allocation concealment

**Figure 1:**
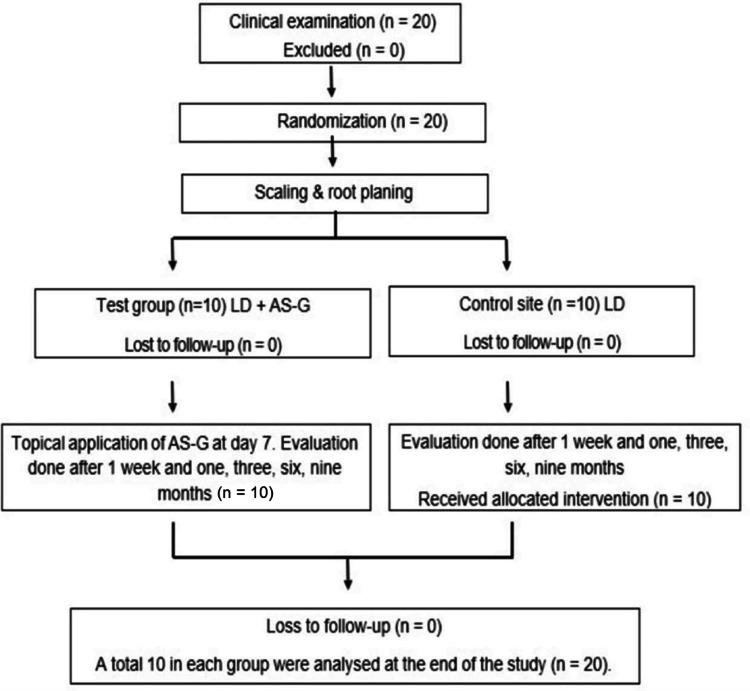
Flowchart showing the participant flow and study design AS-G, ascorbic acid 2-glucoside; LD, laser depigmentation

Eligible participants were randomly assigned to either the control or test group using computer-generated random numbers. Allocation concealment was ensured through sequentially numbered, opaque, sealed envelopes that were opened only at the time of treatment. Randomization was performed by an independent assessor who was not involved in the treatment procedures. Participants were allocated to the control group receiving LD alone or the test group receiving LD followed by topical application of AS-G (LD + AS-G). The participant flow and study design are illustrated in Figure 1.

Blinding

Due to the nature of the intervention, neither the participants nor the personnel administering the intervention were blinded. However, the outcome assessor was blinded to the group allocation to minimize assessment bias. Therefore, the study is more appropriately described as an assessor-blinded study.

Intervention procedures

Prior to the intervention, all participants underwent professional mechanical debridement, including ultrasonic scaling and root planing, to eliminate local irritants and establish a healthy periodontal baseline. Participants were instructed to maintain optimal oral hygiene throughout the study period and were advised to avoid colored foods and mouthwashes on the day of the procedure. Gingival depigmentation was performed two weeks after initial periodontal therapy.

**Figure 2:**
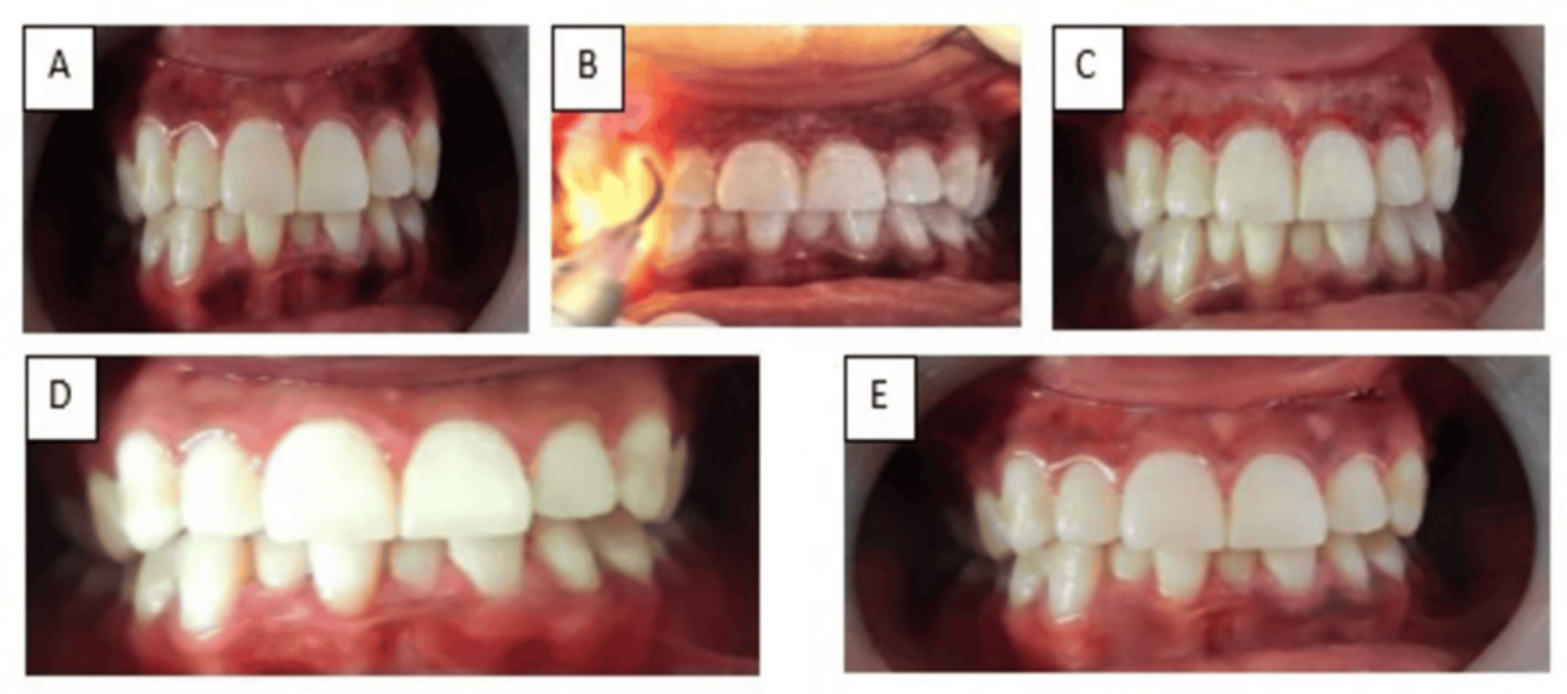
Clinical images of LD (control group) (A) Preoperative image. (B) LD. (C) Immediate postoperative image. (D) One-month follow-up. (E) Repigmentation at nine-month follow-up. LD, laser depigmentation

The treatment site was anesthetized using a topical anesthetic spray (Nummit, ICPA Health Products Ltd, Mumbai, India) containing 15% w/w lidocaine. Gingival depigmentation in both groups was performed using a diode soft-tissue laser (PIOON, Wuhan, China) with a wavelength of 980 nm and power settings between 1 W and 1.5 W. Laser energy was delivered through a 400 μm flexible fiber-optic tip in pulsed contact mode, and the pigmented gingival epithelium was removed using gentle paintbrush-like strokes to achieve uniform ablation [8]. Residual tissue debris was removed with sterile gauze soaked in normal saline. Standard laser safety measures were followed, including protective eyewear for the operator and patient and high-vacuum suction for plume evacuation (Figure 2).

**Figure 3:**
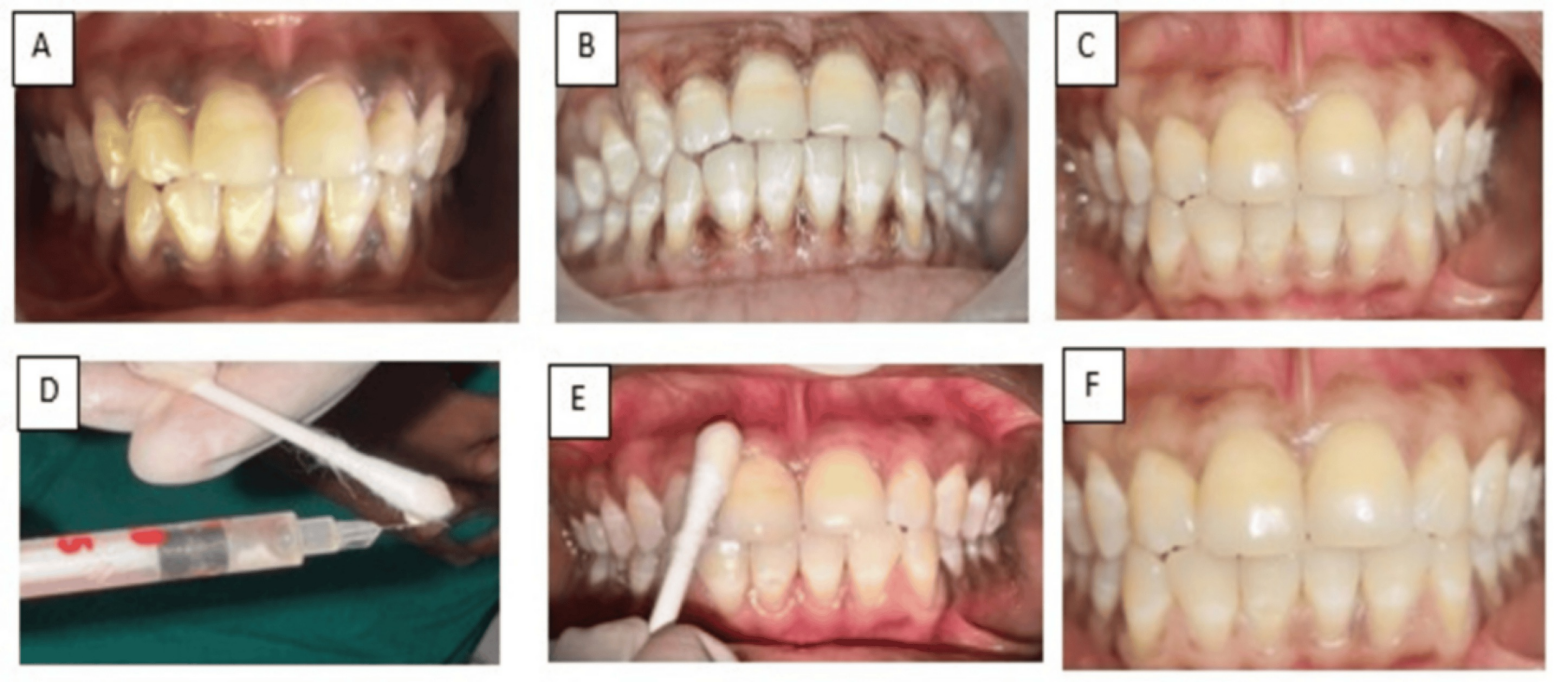
Clinical images of laser + AS-G (test group) (A) Preoperative image. (B) Immediate postoperative image. (C) Seven-day follow-up. (D) AS-G gel. (E) Topical application of AS-G gel. (F) Repigmentation at nine-month follow-up. AS-G, ascorbic acid 2-glucoside

In the test group, topical application of AS-G gel was performed seven days following the depigmentation procedure using sterile cotton buds. The gel formulation consisted of 10% AS-G (w/v) dissolved in distilled water, with propylene glycol and hydroxypropyl methylcellulose as excipients, and was obtained from PerioBiologics LLP (Hyderabad, India). The formulation was stored in individual insulin syringes under refrigerated conditions (2-8°C) and protected from light to maintain stability and sterility (Figure 3).

Outcome measures

Clinical parameters were recorded at baseline and at follow-up intervals of one, three, six, and nine months by the blinded examiner. The primary outcome measure was gingival pigmentation assessed using the Dummett Oral Pigmentation Index (DOPI), a standardized index that evaluates gingival melanin pigmentation on a four-point ordinal scale ranging from 0 (no pigmentation) to 3 (heavy pigmentation). Changes in DOPI scores over time were used to evaluate the effectiveness of topical AS-G in reducing the recurrence of pigmentation following LD.

Secondary outcomes included gingival inflammation, oral hygiene status, and patient satisfaction. Gingival inflammation was assessed using the modified Sulcus Bleeding Index (mSBI), while plaque accumulation was evaluated using the Plaque Index (PI) to monitor oral hygiene as a potential confounding factor. Patient satisfaction with the esthetic outcome was assessed using a 10-cm Visual Analogue Scale (VAS), with scores ranging from 0 (not satisfied at all) to 10 (extremely satisfied). VAS scores were recorded at six and nine months. All indices used in the present study, including PI, mSBI, DOPI, and VAS, are validated and widely used clinical assessment tools reported in the literature [9-11].

Safety monitoring and interim analysis

Participants were monitored throughout the study period for any adverse events or complications associated with the interventions, including postoperative pain, delayed healing, infection, ulceration, or hypersensitivity reactions related to laser therapy or topical AS-G application. All participants were evaluated at each follow-up visit for the presence of any treatment-related complications. All the participants were recalled regularly as per the study protocol, and clinical parameters were recorded at every visit.

Statistical analysis

Statistical analysis was performed using IBM SPSS Statistics for Windows, version 25.0 (released 2017; IBM Corp., Armonk, NY, USA). Descriptive statistics were calculated for all study variables and expressed as mean and SD where appropriate. The normality of the data distribution was assessed using the Shapiro-Wilk test prior to inferential analysis. Since most of the clinical parameters, including the PI, mSBI, and DOPI, are ordinal variables, nonparametric statistical tests were used for their analysis. Intragroup comparisons across different follow-up intervals were performed using the Wilcoxon signed-rank test. Intergroup comparisons for PI and mSBI were carried out using the Mann-Whitney U test. Intergroup comparison of DOPI scores between the test and control groups was performed using the chi-square test. Patient satisfaction assessed using the VAS at six and nine months was analyzed using the independent samples t-test when the assumption of normal distribution was satisfied. All statistical tests were two-tailed, and a p-value of less than 0.05 was considered statistically significant.

## Results

Demographic details analysis

Table 1 shows the comparative analysis of descriptive statistics between control (LD) and test groups (LD + AS-G) based on the variables age and gender. Age-wise distribution of participants was not statistically significant. The distribution of males and females was also not statistically significant.

**Table 1:** Descriptive statistics of both groups Age is expressed as mean ± SD. Gender is expressed as frequency and percentage (n (%)). Total participants per group: n = 10.

Variable	Category	Test	Control
Age (years)	Min	22	23
	Max	28	29
	Mean ± SD	25.0 ± 2.0	26.0 ± 2.0
Gender (n)	Female	6 (60%)	6 (60%)
	Male	4 (40%)	4 (40%)

Intragroup analysis

In Table 2, the results indicated that a statistically significant reduction in PI scores was observed in both the test and control groups at three-, six-, and nine-month intervals, indicating effective plaque control over time. However, for mSBI, no statistically significant changes were observed in the test group at any time interval. In contrast, the control group demonstrated a significant reduction in mSBI only at nine months.

**Table 2:** Intragroup comparison of PI and mSBI at baseline, one, three, six, and nine months in both groups Intragroup comparisons were performed using the Wilcoxon signed-rank test. * p < 0.05 indicates statistical significance. mSBI, modified Sulcus Bleeding Index; PI, Plaque Index

Parameter	Group	Time point comparison	z-Value	p-Value
PI	Test	Baseline to one month	0	1
		Baseline to three months	-2.88	0.004*
		Baseline to six months	-2.8	0.005*
		Baseline to nine months	-2.8	0.005*
	Control	Baseline to one month	0	1
		Baseline to three months	-2.74	0.006
		Baseline to six months	-2.88	0.004*
		Baseline to nine months	-2.8	0.005*
mSBI	Test	Baseline to one month	-1.34	0.18
		Baseline to three months	-1.34	0.18
		Baseline to six months	-0.44	0.66
		Baseline to nine months	-0.81	0.42
	Control	Baseline to one month	-0.21	0.83
		Baseline to three months	-0.54	0.59
		Baseline to six months	-0.44	0.66
		Baseline to nine months	-2.03	0.042*

Intragroup comparison of DOPI scores in both test and control groups

It is revealed in Table 3 that most individuals in the test group had a DOPI score of zero at the follow-up time points. Scores were initially distributed between grades two and three at baseline; however, after one month, four individuals had a score of two and six had a score of three, which was comparable to the baseline distribution. Although pigmentation had nearly entirely disappeared, subsequent assessments revealed that statistical analysis was not possible owing to the uniformity of the data. Similarly, baseline results in the control group were identical to those in the test group. Most control subjects continued to have lower pigmentation scores at one and three months, but a small percentage displayed a score of one by nine months. There was more variance in pigmentation scores, with some control subjects having a score of two. The intragroup differences remained statistically “not computed” despite these modifications, as the distribution was unfit for reliable statistical comparison.

**Table 3:** Intragroup comparison of DOPI in both groups Statistical comparison was not computed due to the absence of variation within the group at that time point. DOPI, Dummett Oral Pigmentation Index

Group	Time point	Score 0	Score 1	Score 2	Score 3	p-Value
Test	Baseline	-	-	4	6	-
	One month	4	0	-	6	-
	Three months	4	0	-	6	-
	Six months	4	1	-	5	-
	Nine months	4	2	-	4	-
Control	Baseline	-	-	4	6	-
	One month	5	1	-	3	-
	Three months	4	2	-	3	-
	Six months	2	4	-	3	-
	Nine months	1	1	4	0	-

Intergroup analysis

The mean scores of PI and mSBI in the test and control groups at baseline and at one, three, six, and nine months are depicted in Table 4. Although there were lower scores in the test group compared to the control group, there was no statistically significant difference at any given time point with respect to PI and mSBI.

**Table 4:** Intergroup comparison of PI and mSBI at baseline, one, three, six, and nine months Values represent mean ranks. Intergroup comparisons were performed using the Mann-Whitney U test. * p < 0.05 indicates statistical significance. mSBI, modified Sulcus Bleeding Index; PI, Plaque Index

Parameter	Time point	Test (mean rank)	Control (mean rank)	z-Value	p-Value
PI	Baseline	11.3	9.7	-0.63	0.526
	One month	11.3	9.7	-0.63	0.526
	Three months	12.6	8.4	-1.65	0.099
	Six months	12.65	8.35	-1.7	0.089
	Nine months	7.55	13.45	-2.29	0.022*
mSBI	Baseline	10.5	10.5	0	1
	One month	9.85	11.15	-0.65	0.516
	Three months	9.4	11.6	-1.03	0.302
	Six months	10.2	10.8	-0.26	0.798
	Nine months	9.8	11.2	-0.57	0.571

Intergroup comparison of the DOPI index at one, three, six, and nine months

The DOPI scores among both groups, as shown in Table 5, indicate that there is no statistically significant difference at baseline, one, three, and six months. The scores at the nine-month evaluation revealed a statistically significant difference between both groups, with higher recurrence at nine months in the control group.

**Table 5:** Intergroup comparison of DOPI index at all time points A chi-square test was used for comparison between groups. * p < 0.05 indicates statistical significance. DOPI, Dummett Oral Pigmentation Index; mSBI, modified Sulcus Bleeding Index; PI, Plaque Index

DOPI time point	Score	Test, n (%)	Control, n (%)	Total, n	χ² Value	p-Value
Baseline	2	4 (40.0%)	4 (40.0%)	8	0	1
	3	6 (60.0%)	6 (60.0%)	12	-	-
One month	0	10 (100%)	8 (80.0%)	18	2.22	0.136
	1	0 (0%)	2 (20.0%)	2	-	-
Three months	0	10 (100%)	7 (70.0%)	17	3.52	0.06
	1	0 (0%)	3 (30.0%)	3	-	-
Six months	0	9 (90.0%)	5 (50.0%)	14	3.81	0.051
	1	1 (10.0%)	5 (50.0%)	6	-	-
Nine months	0	8 (80.0%)	1 (10.0%)	9	11.11	0.004*
	1	2 (20.0%)	4 (40.0%)	6	-	-
	2	0 (0%)	5 (50.0%)	5	-	-

VAS satisfaction evaluation at six and nine months

Patient satisfaction evaluation using VAS at the six-month follow-up revealed no statistically significant difference. There was a statistically significant difference in patient satisfaction in the test group at the nine-month follow-up visit (Table 6).

**Table 6:** Intergroup comparison of patient satisfaction at six and nine months Values are expressed as mean ± SD. Intergroup comparisons were performed using the independent samples t-test. * p < 0.05 indicates statistical significance.

Time point	Group	Mean ± SD	Mean difference	95% CI (lower)	95% CI (upper)	t-Value	p-Value
Six months	Test	4.70 ± 0.48	0.2	-0.27	0.674	0.89	0.388
	Control	4.50 ± 0.52					
Nine months	Test	4.90 ± 0.42	1.3	0.65	1.742	5.21	0.0001*
	Control	3.60 ± 0.69					

## Discussion

Gingival pigmentation most frequently affects the attached gingiva but can also be seen in the alveolar mucosa and interdental papillae. Clinically, pigmentation presents as diffuse or localized areas of brown to black discoloration and can vary greatly in distribution and intensity [12]. The darker appearance seen in gingival hyperpigmentation is usually due to increased melanin accumulation in the basal epithelial layers. While this condition is generally benign and does not indicate any underlying pathology, it can become a cosmetic concern, especially for individuals with high smile lines or increased visibility of the gingiva during speech or expression [13]. Due to increasing awareness of dental aesthetics, many patients seek treatment for gingival hyperpigmentation, even though it poses no health risk. Among these modalities, laser-assisted depigmentation has emerged as a preferred option due to its minimally invasive nature and favorable clinical outcomes [5]. Nevertheless, each technique, whether conventional or laser-based, presents varying degrees of efficacy, healing response, patient comfort, and recurrence rates.

Several studies have highlighted the superiority of laser therapy in achieving aesthetic results. For instance, Jagannathan et al. reported that diode laser treatment was associated with reduced pain, improved patient comfort, shorter procedure time, and accelerated wound healing compared to scalpel and electrosurgery techniques [5]. Similarly, a systematic review by Gul et al. demonstrated significantly lower postoperative pain levels with laser ablation [12].

In addition to conventional methods, the depigmenting potential of vitamin C has received increasing attention in recent years. An important regulator of melanin biosynthesis is ascorbic acid (vitamin C), a potent antioxidant with notable depigmenting properties. Moreover, ascorbic acid contributes to the degradation of existing melanin and interferes with melanosome maturation and transfer, reducing pigmentation at both the cellular and tissue levels [14]. Its role in promoting collagen synthesis, enhancing epithelial healing, and mitigating oxidative stress further supports its use in aesthetic periodontal procedures [15-17]. Shimada et al. observed appreciable improvement in gingival color following topical application of AS-G gel, with results sustained for up to 12 weeks [14]. In a case report, Kabiraj et al. stated that effective gingival depigmentation was achieved using both laser treatment and vitamin C. However, laser treatment yielded better results compared to vitamin C used alone [18].

In the present study, AS-G gel was utilized as an adjunct to diode laser treatment, with the intention of enhancing clinical outcomes and mitigating the likelihood of repigmentation. These findings are consistent with previous research by Shimada et al., who demonstrated similar benefits of ascorbic acid in gingival depigmentation [14]. Sanadi and Deshmukh further corroborated the role of vitamin C in delaying pigment recurrence following treatment [19]. The current study also found a statistically significant difference in DOPI index values at the nine-month follow-up between the control group (LD) and the test group (laser + AS-G). These findings correlate with those of Sheel et al., who observed that topical ascorbic acid, when used as an adjunct to surgical depigmentation, resulted in a noticeable delay in pigment recurrence [20]. Although the precise biological mechanism responsible for repigmentation is not fully understood, the “melanocyte migration theory” proposes that melanocytes from surrounding untreated areas may migrate into the depigmented zones, contributing to recurrence [21].

In addition to clinical assessments, patient-reported satisfaction was also evaluated using a five-point VAS. At the nine-month recall, the test group (laser + AS-G) demonstrated significantly higher satisfaction scores compared to the control group. This suggests that the adjunctive use of vitamin C not only contributed to delayed repigmentation but also improved patients’ perception of aesthetic and therapeutic outcomes. These findings are consistent with earlier studies reporting enhanced healing and cosmetic satisfaction following vitamin C-based interventions [21,22].

Clinical implications

The findings of the present randomized controlled trial suggest that the adjunctive use of topical AS-G following diode laser gingival depigmentation may help reduce the recurrence of physiologic melanin pigmentation and improve esthetic outcomes. This approach may offer clinicians a minimally invasive and cost-effective adjunctive strategy to enhance the stability of depigmentation procedures. However, further studies with larger sample sizes and longer follow-up periods are required to confirm the long-term clinical benefits of this adjunctive therapy.

Limitations

To begin with, the results may not be as broadly applicable due to the small sample size. Second, even though the follow-up period was extended to nine months, it may not be sufficient to assess long-term recurrence rates, particularly as repigmentation can occur over a longer period. Third, the study did not use histological analysis to verify the degree of melanin reduction or cellular alterations; instead, it relied only on clinical indicators (DOPI) and patient-reported outcomes. Therefore, the conclusions of the study should be interpreted as preliminary clinical observations rather than definitive evidence of long-term outcomes. Larger studies with greater sample sizes, extended follow-up periods, and histological assessment would be beneficial to further validate the findings.

In addition to the previously mentioned limitations, the relatively small sample size and single-center design may limit the statistical power and generalizability of the findings. As DOPI is an ordinal outcome measure, the ability to detect subtle differences between groups may also be limited. Furthermore, multiple statistical comparisons across follow-up intervals may increase the risk of Type I error. Therefore, the results should be interpreted with caution and confirmed by larger multicenter studies with longer follow-up.

## Conclusions

Within the limitations of this study, it can be concluded that both laser-assisted depigmentation and laser combined with the topical application of AS-G (vitamin C) are effective in the management of gingival hyperpigmentation. However, the adjunctive use of AS-G demonstrated a statistically significant delay in the recurrence of pigmentation and higher patient satisfaction at the nine-month follow-up. These findings suggest that ascorbic acid may serve as a valuable adjunct in enhancing the aesthetic longevity and patient-centered outcomes of LD procedures. Further studies with larger sample sizes, longer follow-up durations, and histological validation are recommended to substantiate these results and explore the long-term efficacy of vitamin C in preventing gingival repigmentation.
